# Resilient Mechanical Metamaterial Based on Cellulose Nanopaper with Kirigami Structure

**DOI:** 10.3390/nano12142431

**Published:** 2022-07-15

**Authors:** Tadaoki Fujita, Daisuke Nakagawa, Kazuma Komiya, Shingo Ohira, Itsuo Hanasaki

**Affiliations:** Institute of Engineering, Tokyo University of Agriculture and Technology, 2-24-16 Naka-cho, Koganei 184-8588, Tokyo, Japan; s209470u@st.go.tuat.ac.jp (T.F.); s220527q@st.go.tuat.ac.jp (D.N.); s194755x@st.go.tuat.ac.jp (K.K.); s198209t@st.go.tuat.ac.jp (S.O.)

**Keywords:** Kirigami, mechanical, metamaterial, cellulose nanofiber, nanopaper, resilience, flexible, substrate, finite deformation, residual strain

## Abstract

Nanopapers fabricated from cellulose nanofibers (CNFs) are flexible for bending while they are rather stiff against stretching, which is a common feature shared by conventional paper-based materials in contrast with typical elastomers. Cellulose nanopapers have therefore been expected to be adopted in flexible device applications, but their lack of stretching flexibility can be a bottleneck for specific situations. The high stretching flexibility of nanopapers can effectively be realized by the implementation of Kirigami structures, but there has never been discussion on the mechanical resilience where stretching is not a single event. In this study, we experimentally revealed the mechanical resilience of nanopapers implemented with Kirigami structures for stretching flexibility by iterative tensile tests with large strains. Although the residual strains are found to increase with larger maximum strains and a larger number of stretching cycles, the high mechanical resilience was also confirmed, as expected for moderate maximum strains. Furthermore, we also showed that the round edges of cut patterns instead of bare sharp ones significantly improve the mechanical resilience for harsh stretching conditions. Thus, the design principle of relaxing the stress focusing is not only important in circumventing fractures but also in realizing mechanical resilience.

## 1. Introduction

The versatile implementation of wearable devices leads to demand for flexible ones. Flexible devices for sensing applications etc., consist of flexible substrates, and there are typically two categories in this flexibility, i.e., stretchable and not. Substrates based on elastomers such as PDMS show flexibility including stretchability. This is a great advantage for the ultimate pursuit of flexibility. However, this is also a drawback in the implementation of conventional electronic elements onto the substrates. Namely, the elements of a device basically also require the stretchability in accordance with the substrates. Although there are already quite a few examples of such devices [1,2,3], most existing electronic elements on the industrial level are not compatible with stretching. Since the expected applications of flexible wearable devices are diverse [4,5,6,7], the stretchability, e.g., on the molecular scales, are often unnecessary. The necessary space resolution of stretchability depends on the specific applications.

In addition to elastomers such as silicone rubbers, nanopaper [8,9,10,11] made of cellulose nanofibers (CNFs) [12] is also a promising material for flexible device substrates. There have been lots of demonstrations indicating the advantages in printed electronics [13] and related applications [9] because of the fine texture of nanopaper and its transparency [14]. In addition to these advantages, the cellulose nanopapers are environmentally friendly [15] which is often important in field applications. Bio-degradable devices are an emerging concept in this context [16,17,18]. The basic mechanical properties of nanopapers have been extensively studied [19,20,21,22,23,24,25,26,27,28]. Their flexibility in combination with mechanical strength are sufficient for attracting the attention of researchers in the discipline of flexible devices. Because of the fact that nanopapers are papers, the demonstration of Origami was also conducted [29]. Flexibility in terms of bending, and durability in terms of keeping folded states by hand without fracture is a remarkable feature which is shared by ordinary papers such as copy papers.

While Origami is now well-recognized as valuable in the engineering design concept, Kirigami is also recognized as such and provides a useful functionality [30]. Today, the mechanics related to the geometry of Origami and Kirigami functionality is a long lasting important topic in physics and engineering. The cut patterns of dashed lines enable the stretching of materials with poor intrinsic stretchability to the extent that the bending flexibility is sufficient [31,32]. Whereas the Origami concept for engineering design prevails in such a way that includes the dynamics of the rigid body of plates connected by hinges, the Kirigami concept without specific folding part requires the large elastic deformation. In particular, paper-based materials are basically flexible in terms of bending but relatively stiff in terms of stretching. Therefore, paper-based materials implemented with Kirigami structures essentially undergo large bending deformation under loading states for the stretching of the whole structure. This is also an out-of-plane deformation and a kind of bifurcation phenomenon [32]. This is in stark contrast with the elastomer-based mechanical metamaterials realized by the perforation undergoing in-plane large deformation [33,34].

On the one hand, prescribed and usually lasting folded states are required in Origami. On the other hand, resilience from finite and large deformation is desired in the Kirigami metamaterial substrates for the implementation of deformable bodies. Therefore, the stretching flexibility of paper-based mechanical metamaterials realized by Kirigami structure poses a basic question: to what extent does flexibility in terms of bending lead to the sustained deformed state? Since the same materials are employed for different requirements, there must be the design principle to make full use of the fundamental mechanical properties for specific situations. This is even more important when realizing that the Kirigami and Origami mechanism is often simultaneously employed in the same system [30]. The mechanical systems in industries are generally rationally designed based on the principle of mechanics and dynamics. The rational design of Kirigami functionality applicable for diverse situations requires a basic understanding of the mechanical resilience of flexible materials that can also sustain the deformed state.

In this study, we focus on the resilience of nanopapers for flexible substrates equipped with stretching flexibility by the Kirigami structure. Employing basic staggered dashed lines of cut patterns (or “*Amikazari*” patterns) in the Kirigami structure, we evaluate the resilience of nanopapers through iterative tensile test. We evaluate the mechanical resilience by the residual strain. The results reveal the dependence on the maximum strain and the number of iterations in the tensile test, which has not been addressed in the only existing study of Kirigami on nanopaper which focuses on the specific functionality of heat dissipation [35]. Whereas the existing reports on the mechanical properties of nanopapers are rather focused on the stiffness, we report the new aspect of mechanical characteristics. Furthermore, we also show an important design principle for attaining the mechanical resilience as a specific example.

## 2. Methods

### 2.1. Fabrication of Nanopapers from Aqueous CNF Dispersion

**Figure 1:**
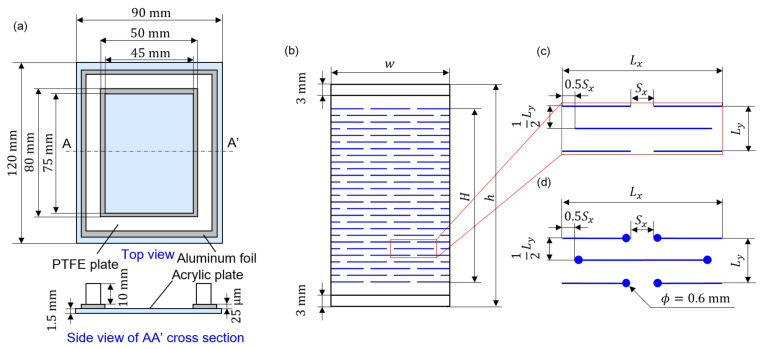
Schematic diagrams of (**a**) the aqueous CNF dispersion container to dry the CNF dispersion, and (**b**–**d**) Kirigami structures: (**b**) The specimen with a cut pattern of staggered dashed lines (or “*Amikazari*) is represented by the blue lines. (**c**) The pattern consists of a unit where the specific set of $L_{x}$, $L_{y}$, $S_{x}$, and *H* determines the pattern, whereas *w* and *h* determine the size of specimen. The gaps of 3 mm on each side of the specimen are in direct contact with the chucks of the tensile testing system. (**d**) Basic and versatile design variation of the Kirigami pattern with the round-edge cutting line is schematically shown. It should be noted here that the design is based on the center lines of a laser processing trajectory and the actual cut lines include round edges have finite widths.

The nanopapers were made from the aqueous dispersion of CNFs. The schematic diagram of the container to dry the CNF dispersion is shown in Figure 1a. The wettability condition is designed by differentiating the materials for the bottom and side walls, each of which is made of acrylic (ACSH-120-90-1.5, MISUMI Corporation, Tokyo, Japan) and polytetrafluoroethylene (PTFE) (PTFE-N-80-20-10 and PTFE-N-70-20-10, MISUMI Corporation, Tokyo, Japan) plates, respectively. Aluminum foil (Aluminum Foil Ultra Thick Type Super Wide, UACJ Foil Corporation, Tokyo, Japan) at the bottom is placed for the easier manual removal of the nanopaper from the acrylic bottom plate. The aqueous CNF dispersion was diluted with purified water (purified water, Kenei Pharmaceutical Co., Ltd., Osaka, Japan) from original concentrations beyond 2 wt% of the TEMPO-oxidized CNF dispersion (I-2SX, DKS Co., Ltd., Kyoto, Japan) using an ultrasonic homogenizer (NR-50M-MT2, Microtech Co., Ltd., Chiba, Japan) for 2 min at 30% of the power under PWM control. The diluted CNF dispersion was vacuumed using inverter diaphragm vacuum pump (NVP-2100V, Tokyo Rikakikai Co., Ltd., Tokyo, Japan) by the multistep decrease in the pressure to 100, 80, 60, 40, 20, and 10 hPa, where each pressure condition was maintained at 10 min. The vacuumed CNF dispersion was placed in the aforementioned container, and then vacuumed again at 20 hPa for 10 min. The vacuumed dispersion in the container was placed in the drying chamber (THR030FA, TOYO ROSHI Co., Ltd., Tokyo, Japan) under the prescribed condition of 40 °C and 50%RH for at least 48 h. The initial CNF concentration $C_{\mathrm{CNF}}$ and volume $V_{L}$ of dispersion in the container are 0.30 wt%, 20 mL for the examination of the unit scale parameter of Kirigami pattern and tensile test conditions, and 0.60 wt%, 20 mL for the examination of the shape of the Kirigami pattern, respectively. The nanopaper fabrication by solution casting and drying presumably leaves residual stress [36] due to the anisotropy of the drying process as the boundary condition [37]. The deviation from the perfectly flat shape due to the residual stress might serve as the origin of the nonuniform deformation in some cases.

### 2.2. Implementation of Cut Patterns of Kirigami Structure by Laser Processing

We employed the basic and well-known Kirigami structure of staggered dashed lines, as schematically shown in Figure 1b,c, to realize the stretching flexibility of the nanopaper specimens. The specific sizes of each part to define the structures are summarized in Table 1. We define the size scaling factor *s* of the Kirigami structure, where dimensional lengths of $L_{x}$, $L_{y}$, $S_{x}$ are proportionally scaled with this value. This variation of *s* is to examine the size effect of the Kirigami structure while keeping the specimen size effectively the same. We used the relatively inexpensive commercial semiconductor-laser processing machine (Podea-01, Podea Co., Ltd., Saitama, Japan) to implement the Kirigami structure on nanopapers. It was actually challenging to use laser processing for nanopapers because the nanopapers are transparent. It is possible to cut the transparent nanopaper with sufficient power, but it is difficult to decrease the cut line width. Therefore, we first drew the cutting line by inkjet printer (LaboJet-600Basic, Microjet Corporation, Nagano, Japan) with a commercial conventional black dye ink (HSM-BK, Seiko Epson Corporation, Nagano, Japan); thereby we cut the black material with relatively lower laser power conditions. We used the inkjet head with an inner diameter ca. 80 μm of the tip part (IJHD-1000, Microjet Corporation, Nagano, Japan). The black lines were drawn by a drop discharge speed of 6.5 m/s and a dot pitch of 200 μm. The laser was irradiated onto the inkjet-drawn black lines on nanopapers that had been dried up. Although the nanopapers were cut from the aluminum frames, they had some deviation from the sufficiently flat shape. As it is problematic in laser processing that requires a precise position in the vertical direction to be consistent with the focus, the nanopapers were placed between flat glass plates (3-2497-20, AsOne corporation, Osaka, Japan). The laser cutting was conducted with a running speed of 1 mm/s, and the irradiation was performed a single time without repetition. The cutting process by laser irradiation is based on the energy transformation into heat. Therefore, there remains some possibility that the edge lines of nanopapers where the laser was irradiated may have experienced some modification of physical properties. However, the precise examination of such an effect is beyond the scope of this study. In this study, we discussed the improvement of mechanical resilience by the essential variation of Kirigami design. Namely, we examined the role of stress focusing due to the Kirigami pattern by the round edge of the cutting line, as shown in Figure 1d.

### 2.3. Evaluation of Resilience by Residual Strain in the Iterative Tensile Test

The specimens of nanopapers with Kirigami structure were loaded on the in-house tensile testing system. The actuator system consists of an AC servo motor (SGMV26-100(X)-0B, Sigmakoki Co., Ltd., Tokyo, Japan), pulse generator (PGC-04-U, Sigmakoki Co., Ltd., Tokyo, Japan), and a driving servo pack (SGDV-2R9EP1A, Yaskawa electric corporation, Fukuoka, Japan). One end of the chuck parts was fixed on the board and the other was connected to the servo motor system. The time series capture of images using a digital camera (DMC-GX8, Panasonic Corporation, Osaka, Japan) with pancake zoom lens (M.ZUIKO DIGITAL ED 14-42mm, F3.5-5.6 EZ, Olympus Corporation, Tokyo, Japan) was conducted from top and side views to examine the deformation characteristics. The conditions of tensile tests are specified by the maximum strain, the number of iterations, and the strain rate. In this article, the strain is evaluated as the nominal value, where the initial length of the specimen is defined as *H* in Figure 1b. The maximum strains were varied as $\varepsilon_{\max}=$ 0.1, 0.2, 0.3, and 0.5. A single cycle of tensile loading started from the original end-to-end distance of the chuck parts to the maximum strain, and then restored the original end-to-end distance again. The total number $N_{c}$ of iterations for a single specimen was $10^{3}$, and the strain rate was ±0.1 $s^{-1}$ in all cases. It should be noted that the maximum strain to be tolerated depends on the number of cutting lines in the tensile direction. We rather assigned the harsh condition from this viewpoint. Accordingly, the number $N_{C}=10^{3}$ of iterations was sufficient for iterative testing. The room temperature and humidity were in the range of 24.2–27.0 °C and ca. 30–50%RH, respectively. The residual strains were evaluated by the side views of digital camera images when the end-to-end distance of the chuck part was at its minimum. The image analysis starts from the Sobel filtering in the two directions sequentially by “Find Edge” option of the software platform ImageJ. Then, the images are binarized with the default criteria of ImageJ. The binarized images are processed in such a way that the vertical position of the paper specimen is determined by the mean of finite thickness. The obtained curve is fitted with 5th-order polynomial, the length $L_{\mathrm{Path}}$ of which is evaluated by the following equation:

(1) $$L_{\mathrm{Path}}=\int_{x_{s}}^{x_{e}}\sqrt{1+{\left(\frac{dy}{dx}\right)}^{2}}dx,$$

where the *x* and *y* directions are defined as the tensile direction and plane normal direction of the initial state of the specimen, and $x_{e}$ and $x_{s}$ indicate both ends of the sampling domain, i.e., $|x_{e}-x_{s}|=H$ (cf. Figure 1b). The smooth fitting instead of pixel-based direct numerical integration is reasonable as the latter leads to an artificially large length by confusion of stepwise macroscopic shape by Kirigami structure.

## 3. Results and Discussion

**Figure 2:**
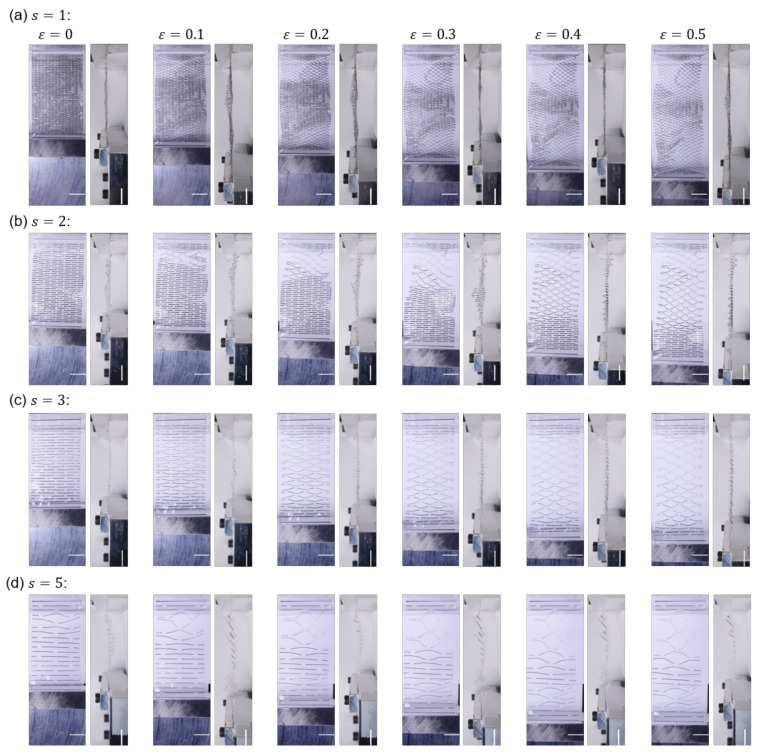
Sequential snapshots in the tensile loading to $\varepsilon_{\max}=0.5$ without iteration (i.e., $N_{c}=1$) for different scale parameters *s*. Six sets of subfigures in each of (**a**–**d**) show the strain ε=0,0.1,0.2,0.3,0.4, and 0.5 from left to right. A unit pair of subfigures for a specific strain value indicate the top and side views of the specimen. The scale bars indicate 10 mm.

**Figure 3:**
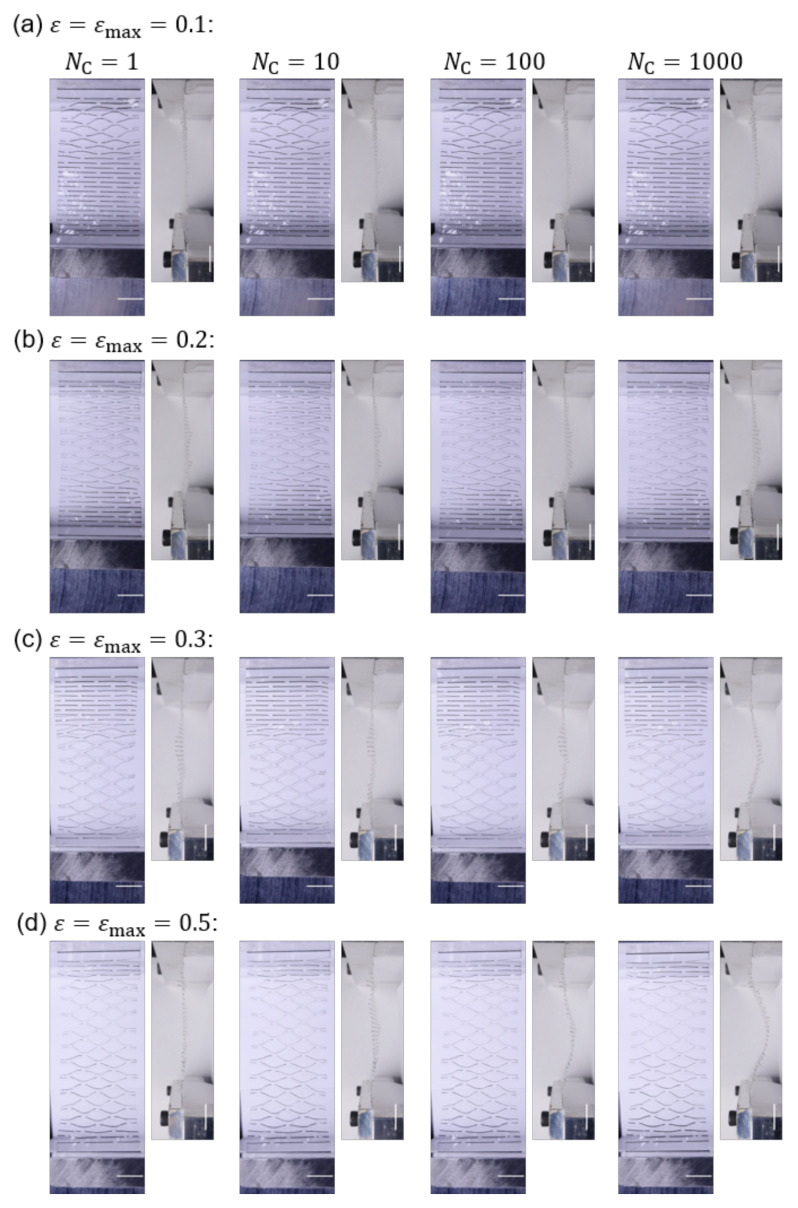
Sequential snapshots in the iterative tensile loading cycles of specimens with s=3 for different maximum strains $\varepsilon_{\max}$. Four sets of subfigures in each of (**a**–**d**) show the number $N_{c}=1,10,100$, and 1000 of tensile loading cycles, respectively—from left to right. A unit pair of subfigures for a specific $N_{c}$ indicate the top and side views of the specimen. It should be noted that top views correspond to the state of $\varepsilon_{\max}$ whereas the side views correspond to the original end-to-end distance state. The scale bars indicate 10 mm.

**Figure 4:**
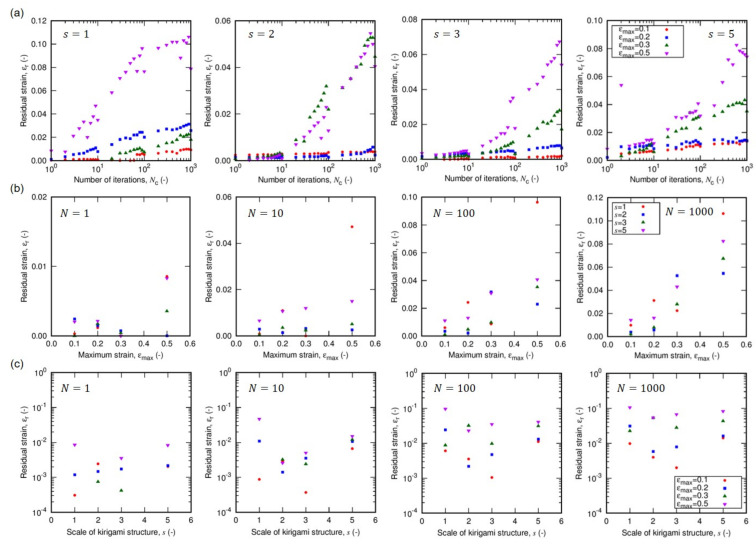
The residual strain as a function of (**a**) the number of tensile loading cycle, (**b**) the maximum strain in a cycle, and (**c**) the unit scale *s* of Kirigami structure. The parameter “*s*” is defined in the Section 2.

Figure 2 and Figure 3 are the sequential snapshots of the actual specimen in the tensile tests. Figure 2 shows the first cycle of the tensile load to $\varepsilon_{\max}=0.5$ for different unit scales *s* of Kirigami structure (cf. Section 2 for definition), and Figure 3 shows the iterative process for different $\varepsilon_{\max}$ for s=3. Figure 4a shows the residual strains $\varepsilon_{r}$ as a function of the number $N_{c}$ of cycles in the iterative tensile test. The subfigures indicate the variation of the unit scale *s* of the Kirigami patterns. The high mechanical resilience of nanopaper implemented with Kirigami functionality can be observed in this figure. The residual strain for a single cycle of tensile test shows $\varepsilon_{r}<0.01$ and $N_{c}=1000$ cycles of the loading keeps $\varepsilon_{r}<0.12$ against $\varepsilon_{\max}=0.5$. Figure 4a also reveals that the moderate maximum strain (i.e., $\varepsilon_{\max}\leq 0.2$) shows remarkably higher resilience compared to the harsh conditions (i.e., $\varepsilon_{\max}\geq 0.3$). There is an overall feature that the iteration of elongation and restoration to the initial end-to-end distance of the specimen cause the increase in residual strain. In addition to $N_{c}$, the maximum strain $\varepsilon_{\max}$ shows a clearly positive correlation with the residual strain, which is further confirmed by Figure 4b. The dependence of $\varepsilon_{r}$ on $\varepsilon_{\max}$ manifests when the loading is iterated.

On the other hand, the dependence of $\varepsilon_{r}$ on the unit scale *s* is hard to reveal in the current setup as shown in Figure 4c. In addition to the fact that the examined range of *s* in this study is obviously huge compared to the thickness of the nanopapers, the tensile tests show rather significant scattering in the absolute values of the residual strains. This large scattering of $\varepsilon_{r}$ (without the clear dependence on the scale parameter *s*) originates from the nonuniform deformation. Figure 2 shows the sequential snapshots of specimens in the first loading of $\varepsilon_{\max}=0.5$ for different *s*. The opening width of cut lines in each specimen is not uniform but there are some parts that keep the almost initial shape, whereas rest of the parts are widely opened by the tensile loading. This nonuniformity is at least partly attributed to the essential characteristics of typical Kirigami-based mechanical metamaterials, which we discuss in the following.

The implementation of staggered dashed-line patterns of Kirigami on a sheet structure is usually intended for the accommodation of substantial elongation for an originally stiff material against tensile loading by making use of bending flexibility. The use of bending flexibility for overall elongation means the induction of out-of-plane deformation. The sheet structures with high flexibility for bending with low flexibility for elongation experience a bifurcation phenomenon in the tensile loading of this staggered dashed-line Kirigami pattern [32]. The in-plane bending deformation shows much higher rigidity than the out-of-plane ones. Therefore, the transition from small in-plane bending to large out-of-plane deformation takes place in the significant tensile loading. Once out-of-plane bending initiates in some parts, the part(s) of the specimen that keep in-plane deformation consequently circumvents a sufficient tensile load because of the flexibility of out-of-plane deforming part(s) serially connected to the tensile direction. Whether this transition from in-plane to out-of-plane bending takes place is sensitive to the initial imperfection and transient asymmetry and/or nonuniformity of the specimen. The loading cannot be uniform on the design as the two ends of the fixed part cannot deform in the direction perpendicular to the tensile direction in the plane of the specimen area.

Nevertheless, sufficient elongation tends to induce more fraction of out-of-plane deformation as shown in Figure 3. Although the out-of-plane bending deformation just after its initiation is highly flexible, the more bending leads to less flexibility (i.e., higher rigidity). Therefore, larger $\varepsilon_{\max}$ corresponds to the state closer to the out-of-plane bending for the entire Kirigami pattern. The cut pattern serially aligned in the tensile direction which effectively becomes softer as the designed number of lines increases. Therefore, there is a balance between the increase in out-of-plane bending parts and the widening of already bending parts. In the latter case, the stress is focused at the edges of cut lines [38]. It is well recognized in engineering and material mechanics that the stress focusing at the edge of the notch is a typical origin of material fracture for ceramics and metals.

Nanopapers are highly flexible against bending deformation, and the bifurcation phenomenon from in-plane to out-of-plane deformation is smooth to the extent that $\varepsilon_{\max}=0.5$ does not lead to the fracture of nanopapers implemented with a reasonable density of Kirigami pattern. The expected applications of nanopaper substrates implemented with Kirigami patterns for tensile flexibility includes the iterative deformation. The iteration $N_{c}=10^{3}$ with $\varepsilon_{\max}=0.5$ does not exhibit fracture either. However, the nonuniform deformation by the mixed structure of flat (or in-plane small deformation) parts and substantial out-of-plane bending parts can cause the remaining deflection at the restored end-to-end distance of the specimen. The fraction of out-of-plane bending parts in the specimen directly affects the level of stress focusing. Therefore, the pursuit of the full restoration of the overall specimen to the flat shape calls for the relaxation of stress focusing at the edges of cut lines.

It should be noted here that there are numerous approaches to improve this mechanical resilience, ranging from the increase in the number of cut lines in some sense, changing the ratio of a specific length design of the unit pattern (instead of the variation of unit scale) to the fundamental variation of the design of the cut line pattern. In this study, we focus on the basic design concept to relax the stress focusing by illustrating a simple approach familiar to the conventional material mechanics for relatively hard materials. Namely, relaxing the sharpness of the cut line edges by locating round edges. This solution example is important due to its versatility/universality. The cutting by knives or lasers usually leaves sharp ends of cut lines. Therefore, the application of the round edge is not limited to the staggered dashed lines or straight-line patterns but it can be applied to arbitrary cut patterns when necessary.

**Figure 5:**
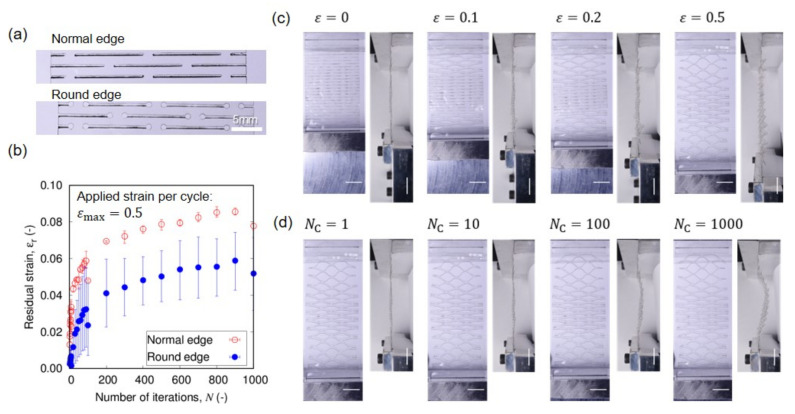
The characteristics of Kirigami design based on the cut lines with round edges: (**a**) Digital camera images of cut lines based on the sharp edge and the round edge on nanopapers. (**b**) Comparison of residual strain by Kirigami patterns with round edge and normal edge. The scale of Kirigami patterns is s=3 and the maximum strain is $\varepsilon_{\max}=0.5$. The error bars indicate the maximum and minimum values in the three trials of different specimens. (**c**,**d**) Sequential snapshots in the tensile tests of Kirigami patterns with round edges with scale parameter s=3, where (**c**) the tensile loading to $\varepsilon_{\max}=0.5$ without iteration (i.e., $N_{c}=1$), and (**d**) iterative tensile loading cycles with $\varepsilon_{\max}=0.5$. The unit pair of subfigures for a specific strain value in (**c**,**d**) indicate the top and side views of the specimen. It should be noted that side views in (**c**) indicate the tensile state and those in (**d**) indicate the state of the original end-to-end distance, while top views in (**d**) indicate the state of $\varepsilon_{\max}$. The scale bars indicate 10 mm.

The round edges of cut lines are designed in such a way to be schematically shown in Figure 1d and actually implemented as shown in Figure 5a. The diameter of the edge is roughly three times that of the cut line. The larger radius of the curvature would enable more relaxation of the stress focusing, but this representative design keeps the advantage of the negligible waste area compared to the cut lines. This small variation of the cutting edge leads to a qualitatively similar behavior of elongation that leaves nonuniformity as shown in Figure 5c,d. Nevertheless, the mechanical resilience is significantly improved as shown by the smaller residual strains for all the tensile iteration cycles in Figure 5b. Thus, the relaxation of stress focusing is one of the important approaches to enable the better mechanical resilience of flexible substrates based on nanopapers. In contrast to the Origami based on the folding, i.e., the strain localization, the mechanical resilience of Kirigami is attained by the nonlocalized prevailing strains. Further improvement of mechanical resilience is expected by more uniform deformation. The current nonuniformity of deformation is at least partly attributed to the gap of loading in the bifurcation from in-plane deformation to out-of-plane ones.

## 4. Conclusions

We examined and revealed the mechanical resilience of cellulose nanopaper implemented with a Kirigami structure by the iterative tensile tests. The nanopapers are basically flexible against bending but rather stiff against tensile loads. Thus, substantial tensile flexibility is enabled by the implementation of a Kirigami structure. The effective tensile flexibility works in such a way that elongation is accommodated by the out-of-plane bending. The nonlinearity of the bifurcation phenomenon from in-plane to out-of-plane bending transition causes the nonuniformity of the strain distribution of Kirigami cut-line units and the scattering of testing results. This nonuniformity contributes to the stress focusing and residual strain. The iterative tensile test reveals the characteristics that has not been clearly addressed. The residual strain increases with larger maximum strain per single elongation and restoration cycle, and the number of iterations enhances this dependence on the maximum strain. The testing condition is rather harsh as the maximum strain is in the range of 0.1–0.5 and the goal of this study is not the champion condition but the general characteristics with an important design principle for the mechanical resilience. We have shown that the round edges of the cut pattern significantly improve the mechanical resilience by the relaxation of stress focusing. This approach is versatile since the sharp edges of cut lines are ubiquitous in the Kirigami designs. Thus, the approach to relax the stress focusing is also advantageous for the mechanical resilience of paper-based soft materials, in addition to the circumvention of a fracture in the hard materials.

## Figures and Tables

**Table 1:** The combination of parameters of $s,w,h,H,L_{x},L_{y}$, and $S_{x}$ of the design of the Kirigami structure. The definitions of parameters are schematically shown in Figure 1b,c. *H* is defined as the initial specimen length relevant to the evaluation of strains. *s* indicates the ratio of the unit size of the Kirigami structure to the standard one in the experiments without the variation in shape. *h* indicates the whole specimen length.

*s* (-)	*w* (mm)	*h* (mm)	*H* (mm)	$L_{x}$ (mm)	$L_{y}$ (mm)	$S_{x}$ (mm)
1	32.0	60.0	49.2	4.2	1.2	0.6
2			48.0	8.4	2.4	1.2
3			46.8	12.6	3.6	1.8
5			48.0	21.0	6.0	3.0

## Data Availability

The data that support the findings of this study are available upon reasonable request from the authors.
